# Detection of CRISPR-mediated genome modifications through altered methylation patterns of CpG islands

**DOI:** 10.1186/s12864-020-07233-2

**Published:** 2020-12-02

**Authors:** M. Heath Farris, Pamela A. Texter, Agustin A. Mora, Michael V. Wiles, Ellen F. Mac Garrigle, Sybil A. Klaus, Kristine Rosfjord

**Affiliations:** 1 The MITRE Corporation, 7515 Colshire Drive, McLean, Virginia, 22102 USA; 2 The Jackson Laboratory, Technology Evaluation and Development, Bar Harbor, ME USA

**Keywords:** CRISPR genome editing, Homology-directed repair, Non-homologous end-joining, Epigenetic modification, CpG island, Methylation variance, Statistical variance detection

## Abstract

**Background:**

The development and application of CRISPR technologies for the modification of the genome are rapidly expanding. Advances in the field describe new CRISPR components that are strategically engineered to improve the precision and reliability of CRISPR editing within the genome sequence. Genome modification using induced genome breaks that are targeted and mediated by CRISPR components leverage cellular mechanisms for repair like homology directed repair (HDR) to incorporate genomic edits with increased precision.

**Results:**

In this report, we describe the gain of methylation at typically hypomethylated CpG island (CGI) locations affected by the CRISPR-mediated incorporation of donor DNA using HDR mechanisms. With characterization of CpG methylation patterns using whole genome bisulfite sequencing, these CGI methylation disruptions trace the insertion of the donor DNA during the genomic edit. These insertions mediated by homology-directed recombination disrupt the generational methylation pattern stability of the edited CGI within the cells and their cellular lineage within the animal strain, persisting across generations. Our approach describes a statistically based workflow for indicating locations of modified CGIs and provides a mechanism for evaluating the directed modification of the methylome of the affected CGI at the CpG-level.

**Conclusions:**

With advances in genome modification technology comes the need to detect the level and persistence of methylation change that modifications to the genomic sequence impose upon the collaterally edited methylome. Any modification of the methylome of somatic or germline cells could have implications for gene regulation mechanisms governed by the methylation patterns of CGI regions in the application of therapeutic edits of more sensitively regulated genomic regions. The method described here locates the directed modification of the mouse epigenome that persists over generations. While this observance would require supporting molecular observations such as direct sequence changes or gene expression changes, the observation of epigenetic modification provides an indicator that intentionally directed genomic edits can lead to collateral, unintentional epigenomic changes post modification with generational persistence.

**Supplementary Information:**

The online version contains supplementary material available at 10.1186/s12864-020-07233-2.

## Background

The emergence of rapid genome editing methodologies facilitated by CRISPR/Cas9 [1] and similar technologies has opened the ability to generated stable and precise edits within animal models [2], accelerating the rate of disease research and creating a shorter path toward disease treatments, therapeutic applications, and agricultural improvements. Further, these technologies are poised to be used in human therapeutic applications. In regard to mice, CRISPR/Cas9-mediated edits are applied at the zygote stages introducing various genomic mutations [3, 4], resulting in the development of numerous mouse models. Tissue- and cell-targeted applications like somatic gene therapy can also deliver genetic modification components to the respective targets via molecular and physical carriers, including liposomes [5–7], adeno-associated virus [8–10], lentivirus [11], and electroporation [12]. These genome modification approaches have their respective challenges and limitations; however, these and other chemical and physical transfection methodologies have successfully generated representative mouse models of human disease, using CRISPR components. Using similar approaches, many other species, including humans [13], have been genetically modified by CRISPR and CRISPR-like systems.

At the application of CRISPR and similar genome editing technologies, the genome is typically edited by introducing a DNA break at the target site, leading to non-homologous end joining (NHEJ) and resulting in indels. When donor DNA is present, it can be incorporated seamlessly into the break mainly by homology-directed repair (HDR). With the rise and expansion of these technologies, the rate and precision of the complete introduction of genomic modifications are increasing, as are the diversity of species that can be edited by them. The incorporation of specific edits into the genome using CRISPR is mediated by the insertion of genomically homologous sequences flanking a non-homologous insertion sequence through HDR [3, 14, 15], by blunt-end insertions and insertion/deletion (indel) mutations through NHEJ repair mechanisms [3], or by fusions of deactivated Cas9 with effector molecules resulting in point modifications by base editors [16] and reverse transcription-mediated sequence insertion by prime editors [17]. Each method provides its advantages and disadvantages; however, HDR methods have to-date proven most versatile and efficient for precise introduction of larger insertions, while NHEJ, which is slightly serendipitous in its outcome, is used extensively as a gene disruption tool.

In general, the genomes of vertebrates are depleted of CpG dinucleotides; however, discrete regions of high G/C content exist across the genome, containing a high frequency of CpG dinucleotides. These clusters of CpG-rich regions are known as CpG islands (CGIs) [18]. Within the mammalian system, CpG locations are available for targeted methylation by the cellular machinery, and the genomic methylation patterns established within CGIs are reset and reestablished during early embryogenesis [19, 20]. CGIs are typically located in gene promoter regions, gene enhancer regions, or within genes themselves and are thought to play an essential role in gene expression regulatory function [21]. DNA methyltransferases, DNMT3A and DNMT3B in combination with DNMT3L, establish the initial DNA methylation pattern at CpG locations. The pattern is maintained by DNMT1 and associated proteins through cell divisions and during the life of the cell [22]. De novo methylation during early development regulates the expression of developmental stage-critical genes and is facilitated by DNMT3a and DNMT3b [23]. Although influences like environmental exposures and tissue inflammation can affect the methylome pattern as an animal ages [24], CGIs with dense occurrences of CpG bases are usually maintained in a hypomethylated state in the vertebrate genome, while across the majority of the genome, occurrences of CpG are less frequent and tend to be in a methylated state [22]. Commonly, CGIs are maintained in an unmethylated state and play a role in the control of gene expression. Induced changes to this preserved methylation pattern within the epigenome can be affected by environmental factors and can have transgenerational effects [25]. Directed reprogramming of the epigenome can replace the epigenetic signatures acquired during development, or imposed by the environment [26]. Further, it has been shown that reprogramming of the cell epigenome using technologies like CRISPR has utility in directing the epigenetic reprogramming of cell fate and function [27–29]. These directed epigenetic changes are thought to be able to persist across generations with progeny displaying the modifications implemented in the parental strains [26]; however, many of the fundamental mechanisms of controlling CpG methylation and their impacts are not fully understood. As previously reported, modifications within the epigenome can be transmitted from parent to offspring and have been shown to be maintained over multiple generations [30]. These changes in the methylome pattern of the genome can induce unexpected changes in the regulation of cellular pathways and characteristics. Through the regulation of CGI methylation patterns, the cell controls or influences important processes, including cellular differentiation, X chromosome inactivation, genomic imprinting, skeletal muscle regeneration, transgenerational influences, and aging processes [30]. An unintended deregulation of regulatory elements in these processes can have unpredictable consequences for the cell and hence the organism [26, 31].

In this study, we highlight perturbations of the epigenome induced by the incorporation of donor DNA during CRISPR genome editing. The result of this sequence replacement or insertion is an alteration of the methylation patterns of CGIs within the region of the genome edit. This methylome change is linked to the footprint of the inserted donor DNA and is inheritable across generations. CRISPR edits leveraging HDR mechanisms and donor homology arms that are localized around CGIs modify the methylation patterns of these CGIs, resulting in increased methylation of the CGIs within the region. These methylation changes within the CGIs are stable and persist across generations. These methylome perturbances were not observed in indel edits using NHEJ, where resulting CGI methylation patterns remained unmodified and consistent with the base unedited state of CGI methylation in the same genetic background.

These observations are impactful for the implementation of genome edits and the development of new tools to study CRISPR/Cas-mediated edits and CGI regulation mechanisms. Further, modifications to potential regulatory elements have implications for the application of these type genomic edits for gene therapies. Influencing the gene expression regulatory functions of CGI methylation patterns, introduction of exogenous donor DNA that could inadvertently methylate neighboring CGIs could affect gene expression regulation of the modified gene or surrounding genes. The ability to observe and direct changes in CGI methylation patterns using CRISPR/Cas9 components also provides the opportunity for molecular tool development. The observation of these CGI methylation changes represents one mechanism to detect applications of CRISPR technology to induce site-directed edits, leaving genomic scars that echo through generations orthogonal to naturally occurring methylation patterns. Further, leveraging the multiplexing abilities of CRISPR technology, modification of one or many CGIs in this manner provides a tool for tracking the functional influence of CGI disruptions within an animal and across generations in either a tissue-specific or systemic manner.

Here we describe a statistical methodology for the detection of CGI methylation variance as a result of CRISPR-mediated genomic editing and HDR donor DNA substitution. We find unexpected transgenerational localized methylation changes upon the addition of non-native donor DNA to the mouse genome. These changes could lead to unintentional changes in gene expression and requires deeper research to understand the local methylation modifications and the unintended influences they may have in human and agricultural applications. Further, these methylation perturbances provide a molecular marker for the characterization of genome edits incorporated into the genome with persistence across generations.

## Results

### Mouse methylome evaluations

The methylomes of CRISPR-edited mice were evaluated using whole genome bisulfite sequencing to determine the modifications induced within the methylome by the CRISPR editing process. Three edited strains and their controls, described in Table 1, were evaluated. The epigenome modifications observed within the mouse strains were secondarily introduced into the genomes by homology-directed repair CRISPR edits. Findings described within in the methodology here indicate an increase in methylation observations within the regions of the adopted homology arms.

**Table 1:** Mouse strains

Abbreviated Name	JAX #	Genomic Modification Details	Mouse Strain
Control 1	005304	Wild-type parental strain	C57Bl/6NJ
Control 2	000664	Wild-type parental strain	C57Bl/6 J
HDR1	028551	CRISPR-mediated HDR insertion using the pRosa-Cas9 targeting vector with an 11-kb insert flanked by *Rosa26* locus homology regions, extending 1 kb upstream and 4 kb downstream from the *Xba*I site within exon 1. The insert contained a codon-optimized *Cas*9 gene linked to an IRES-GFP.	C57B1/6 J*-Gt(ROSA)26Sor*^em1(CAG-cas9*,-EGFP)Rsky^/J
HDR2	028543	CRISPR-mediated HDR insertion of a 2005-bp donor DNA containing eGFP with a poly A signal in an inverted orientation at the start codon of the *Rxfp*3 locus. The original insertion vector had homology arms of 1 kb, flanking the insertion sequence.	C57BL/6NJ*-Rxfp3*^*em2J*^*/*J
NHEJ1	028573	CRISPR-mediated NHEJ insertion of a 48 base pair duplex DNA oligo with no homology arms into the *Rosa26* locus. During endogenous DNA repair, a serendipitous indel of −3 bp and + 6 bp occurred.	C57BL/6 J*-Gt(ROSA)26Sor*^*em2Mvw/Mvw*^

**Fig. 1:**
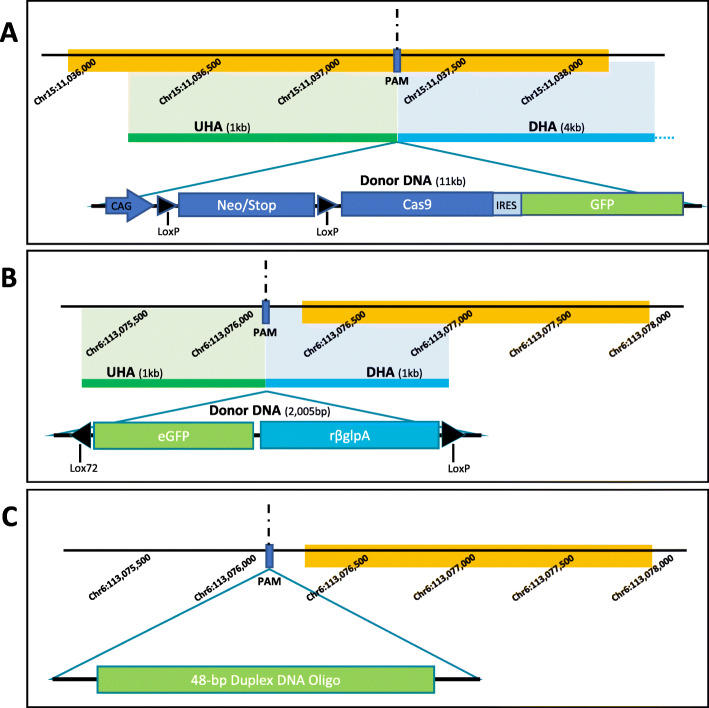
Mouse strain edit sites. The methylation patterns of three CRISPR-edited animal strains were evaluated in this study, describe in detail in Table 1. Strain HDR1 (**a**) contains an 11-kb insert and was recombinantly inserted into the *Rxfp*3 locus of the mouse genome using a 1-kb upstream homology arm (UHA) and a 4-kb downstream homology arm (DHA). The protospacer adjacent motif (PAM) site was located at Chr15:11,037,280. Strain HDR2 (**b**) contains a 2005-bp insert and was recombinantly inserted into the *Rosa26* locus of the mouse genome using a 1-kb UHA and a 1-kb DHA. The PAM site was located at Chr6:113,076,025. Strain NHEJ1 (**c**) contains a 48-bp duplex DNA oligonucleotide fragment incorporated into the *Rosa26* locus by non-homologous end-joining. The PAM site was located at Chr6:113,076,025. For each figure, the yellow bar indicates the CGI location

The effects of homology-directed repair were evaluated using two CRISPR-edited strains (HDR1 and HDR2) obtained from The Jackson Laboratory. HDR1 (028551; C57B1/6 J*-Gt(ROSA)26Sor*^em1(CAG-cas9*,-EGFP)Rsky^/J) (Fig. 1a) contained a CRISPR-mediated homology-directed repair insertion using the p*Rosa*-Cas9 targeting vector with an 11-kb insert flanked by *Rosa26* locus homology regions, extending 1 kb upstream and 4 kb downstream from the *Xba*I site within exon 1. The insert contained a codon-optimized *Cas9* gene linked to an IRES-GFP. HDR2 (028543; C57BL/6NJ*-Rxfp3*^*em2J*^*/*J) (Fig. 1b) contained a CRISPR-mediated homology-directed repair insertion of a 2005-bp donor DNA, containing eGFP with a poly-A signal in an inverted orientation at the start codon of the *RXfp3* locus. The original insertion vector had homology arms of 1 kb, flanking the insertion sequence. The effects of non-homologous end-joining repair were evaluated using an edited strain (NHEJ1) from The Jackson Laboratory. NHEJ1 (028573; C57BL/6 J*-Gt(ROSA)26Sor*^*em2Mvw/Mvw*^) (Fig. 1c) contained a CRISPR-mediated non-homologous end-joining repair insertion of a 48-bp duplex DNA oligo with no homology arms into the *Rosa26* locus. During endogenous DNA repair, a serendipitous indel of − 3 bp and + 6 bp occurred. Control strains included wild-type parental strains Control 1 (005304; C57Bl/6NJ) and Control 2 (000664; C57Bl/6 J).

### Whole genome bisulfite sequencing for a genome-wide evaluation of CpG and CGI methylation patterns

**Fig. 2:**
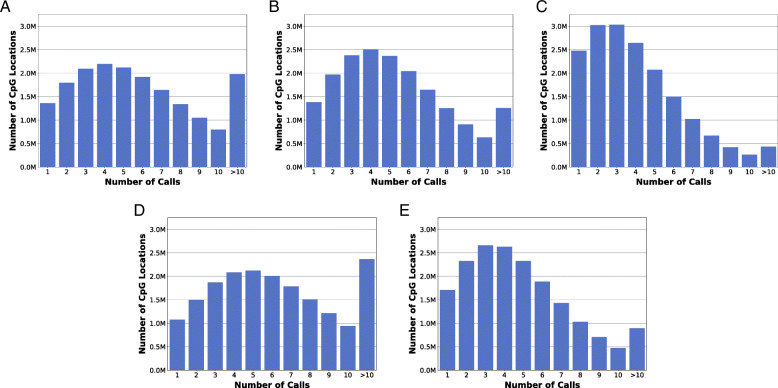
Genome-wide sequence depth of CpG locations. WGBS was used to evaluate CpG methylation profiles within the genome of each edited and control animal. The distribution of the number of CpG calls observed is shown for HDR1 Animal (**a**) with 10,835,815 CpG locations with ≥5 calls, HDR2 Animal (**b**) with 10,109,462 CpG locations with ≥5 calls, NHEJ1 Animal (**c**) with 6,382,801 CpG locations with ≥5 calls, Control 1 Animal (**d**) with 11,948,365 CpG locations with ≥5 calls, and Control 2 Animal (**e**) with 8,754,364 CpG locations with ≥5 calls. The numbers of CpG locations are reported in millions (M)

To determine differential methylation of CpG islands across the genomes of the evaluated animals, we sequenced the genome-wide methylation patterns of CpGs using whole genome bisulfite sequencing (WGBS). The WGBS called methylation levels at CpG dinucleotide locations with high coverage (≥ 5 calls) for each animal. The resulting sequenced methylomes were sequenced at a high depth of coverage across the genome, allowing all CpG site evaluations to contain a minimum of five calls (Fig. 2). The epigenomes of HDR1 and HDR2 were sequenced at an average of 13× CpG coverage with a total of 758,846,055 read pairs and at an average of 11× CpG coverage with a total of 798,607,892 read pairs, respectively. NHEJ1 was sequenced at an average of 8× CpG coverage with a total of 665,977,690 read pairs. The epigenomes of Control 1 and Control 2 were sequenced at an average of 13× CpG coverage with a total of 894,080,435 read pairs and at an average of 10× CpG coverage with a total of 883,336,536 read pairs, respectively. Each animal epigenome had a bisulfite conversion rate of 99%.

An algorithm was developed base on CGI parameters defined by Gardiner-Garden and Frommer [18]. The algorithm called 90,953 enriched CpG regions that were termed CGIs within this study across the mouse genome, having a genome-wide average CGI length of 449.50 base pairs, an average CpG site content of 28.13, and an average CG composition of 55.75%. The identified CpG sites within the CGI ranges in each autosomal chromosome were used in statistical evaluations for methylation variance (S1 Table).

**Fig. 3:**
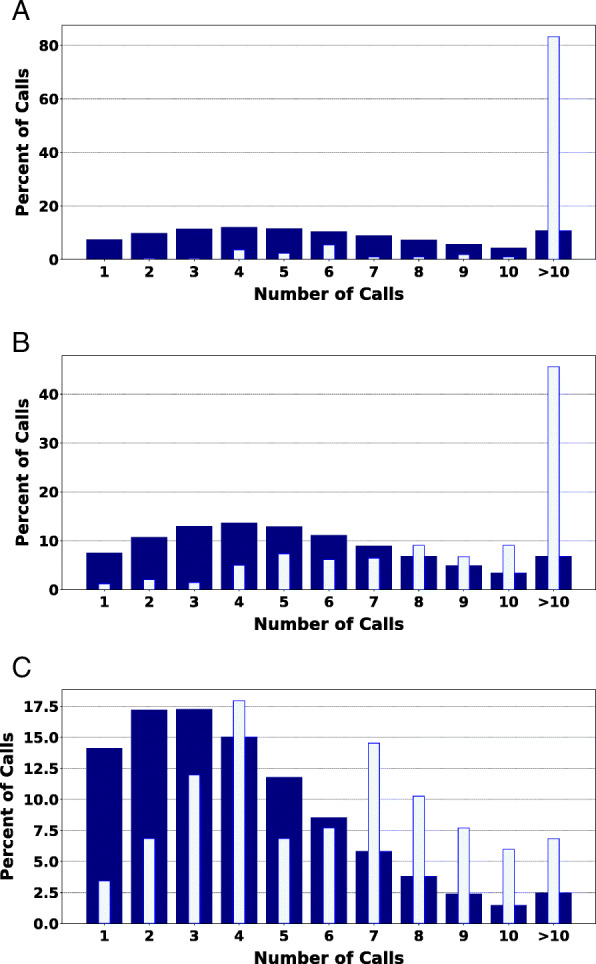
Genomic and CRISPR-edited CGI methylation depth of call percentage. The percent of CpG calls within the respective CRISPR-edited sites for the modified animals exceeds that observed within the genome. For each call depth, the percentage of CpG calls are plotted for the CRISPR-edited CGI (light blue) as related to the genome (dark blue) for HDR1 Animal (**a**), HDR2 Animal (**b**), and NHEJ1 Animal (**c**)

The comparison of the average depth of CpG location bisulfite sequencing (BS-Seq) calls for the genome and the respective CRISPR-edited CGI range for each experimental animal indicates a CpG depth of coverage in the CGIs that is greater than the average across the genome with an approximate 20% increase in calls at a depth of five or more, lending support to the confidence of the methylation observations at the CpG locations of the CGIs (Fig. 3). The CpG locations called in the edited CGIs were sequenced at a depth of five calls or more for 95.48% (211 of 221) of CpG locations in HDR1 Animal, 90.35% (309 of 342) of CpG locations in HDR2 Animal, and 59.83% (70 of 117) of CpG locations in NHEJ1 Animal. Further, calls deeper than 10 calls for CpG location were 83.26% (184 of 221) for HDR1 Animal, 45.61% (156 of 342) for HDR2 Animal, and 6.84% (8 of 117) for NHEJ1 Animal.

### Statistical selection of CGIs with modified methylation patterns

**Fig. 4:**
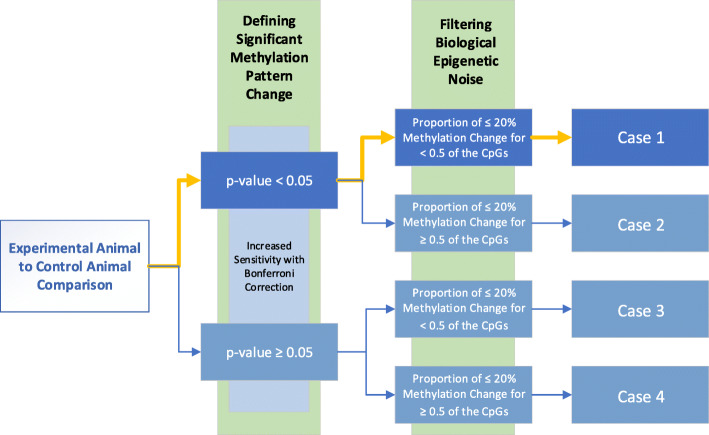
Statistical filter process. The stepwise process for ranking and categorizing the CpG methylation patterns of genomic CGIs permitted the ranking of CGI changes between edited and control animal CGIs. Significantly changed CGIs followed a path (yellow bars) toward Case 1 based on significant *p*-value changes with Bonferroni correction and filtered beyond biological epigenetic noise

The statistical filters (Fig. 4) were empirically selected and applied to the epigenetic profiles of CGIs across the mouse genome to identify CGIs with statistically significant positive change from the control animal (Case 1). Correcting for the large number of evaluations made across the total of the CGIs within each animal, the *p*-values for each CGI range within the given animal comparison were calculated with a Bonferroni correction, where α equals 0.05 divided by the total number of CGI comparisons. Only a small portion of CGIs in each animal displayed significant epigenetic change from the CGIs of the Control 1 Animal (Table 2) with each experimental comparison closely matching the results of control comparisons. HDR1 Animal displayed 0.052% CGI methylation change (37 of 71,120); HDR2 Animal displayed 0.037% CGI methylation change (26 of 69,334); NHEJ1 Animal displayed 0.018% CGI methylation change (11 of 60,450); and Control 2 Animal displayed 0.044% CGI methylation change (29 of 66,545). These similar *p*-value results across all animals with minimal outlying CGIs indicate reproducible comparisons of the large number of CGI observations, allowing enrichment for the targeted change of the edited CGI methylome. The surviving CGIs were reduced further through the application of an observed biological noise filter. This filter was applied to account for minor methylation changes that could be part of normal cellular processes or introduced experimental biases. The surviving CGIs were enriched for those displaying methylation changes beyond the determined 20% threshold for change. HDR1 Animal displayed 0.027% surviving CGIs (19 of 71,120); HDR2 Animal displayed 0.013% surviving CGIs (9 of 69,334); NHEJ1 Animal displayed 0.008% surviving CGIs (5 of 60,450); and Control 2 Animal displayed 0.017% surviving CGIs (11 of 66,545). The sites edited using donor DNA and homology-directed repair mechanisms for genomic incorporation (HDR1 Animal and HDR2 Animal) each displayed significant methylation changes that placed them in the Case1 observations. The edit site of NHEJ1 Animal did not show significant change, highlighting the CGI sequence was not substituted for donor DNA during the NHEJ mutation process.

**Table 2:**
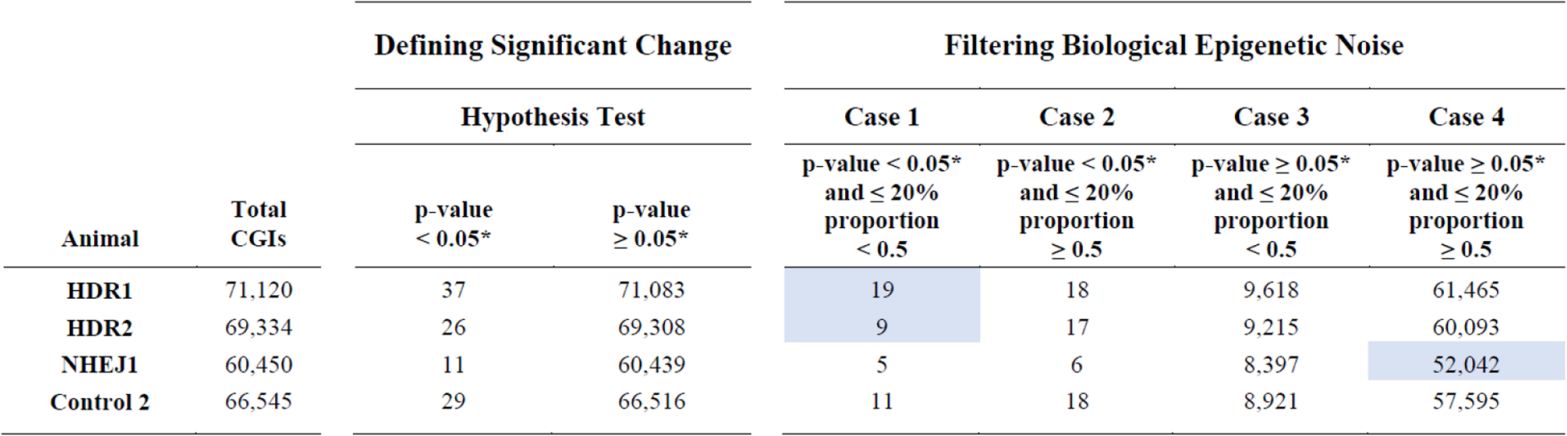
Statistical and epigenetic noise filtering of experimental and control CGI methylome comparisons

The table summarizes the results of evaluating Animals HDR1, HDR2, NHEJ1, and Control 2 against Animal Control 1 using the methodology for identifying significant changes in methylation patterns for CGIs defined in Fig. 4. *Note: The α level of 0.05 was adjusted using the Bonferroni method for each comparison; the adjusted α = 0.05/Total CGIs. Each animal was compared against Control 1 Animal. The blue highlight indicates the Case containing the CRISPR-edited CGI for the respective animal

### CRISPR edits mediated by HDR display methylation profiles at CGIs with distinctive increases in methylation

**Fig. 5:**
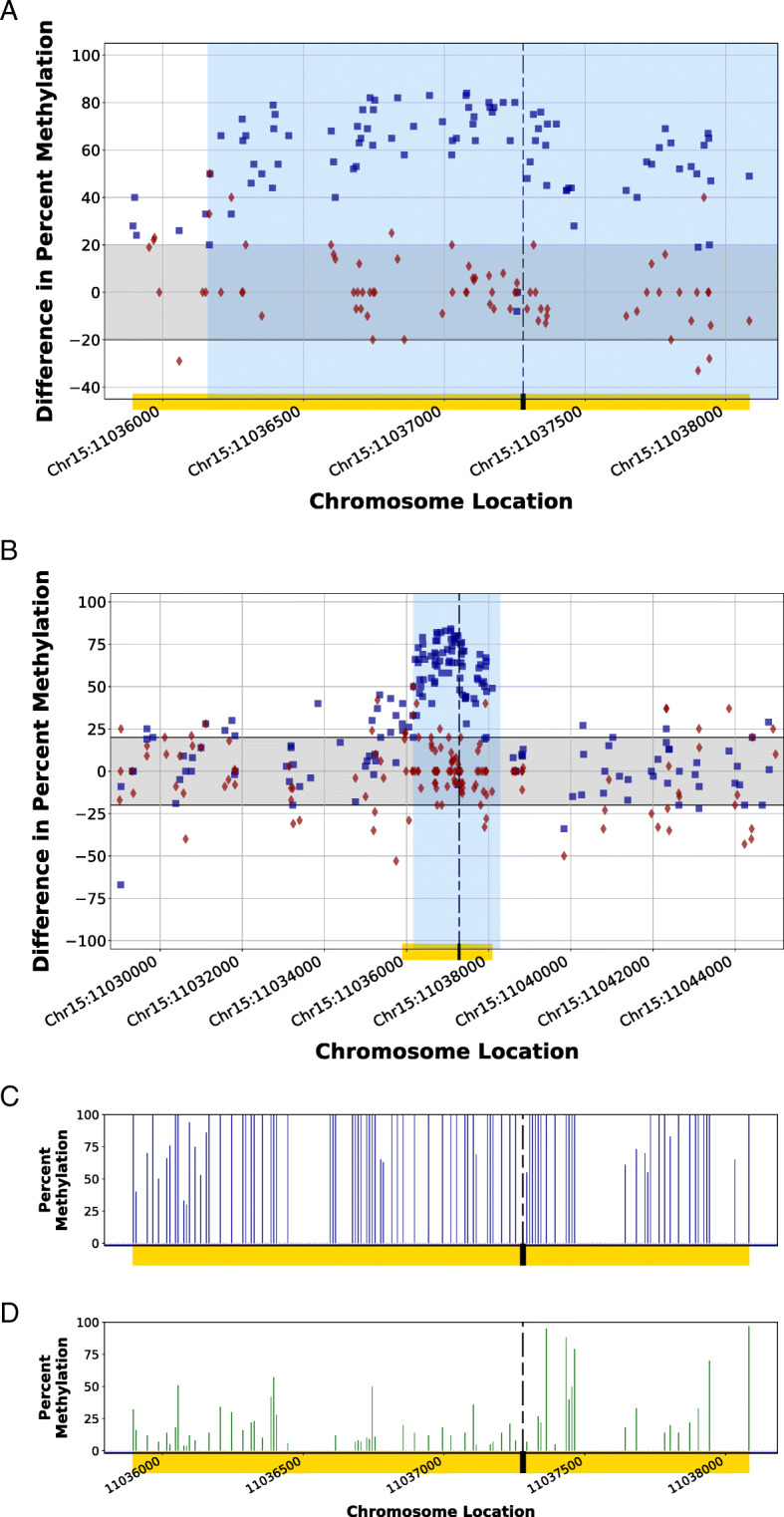
Methylomic difference of a completely edited CpG island within the genome of the HDR1 Animal using homology-directed repair in a CRISPR-mediated edit. Homology arms of a synthetic DNA fragment (region shaded in blue) in a homology-directed repair of a CRISPR-mediated genomic cut spanned a CpG island (CGI; Chr15:11,035,894–11,038,084; yellow bar) within the genome of HDR1 Animal, resulting in a destabilized methylation pattern (blue squares) as compared to the Control 1 Animal methylation patterns at the same location (red diamonds). Figure 4a illustrates the localized methylation variance introduced at the CGI, while Fig. 4b illustrates the comparison of the modified region to flanking endogenous genomic regions by displaying the variance of methylation patterns for 7000 bp upstream and downstream of the CGI. The methylation patterns of regions not influenced by the incorporation of donor DNA during the CRISPR edit displayed a methylation pattern similar to the control. The gray band indicates considered biological epigenetic variance (+/− 20% change). The dashed line indicates the protospacer adjacent motif (PAM) location of the targeted CRISPR cut site. Synthetic homology arms corresponded to genomic sequence 1000 bp upstream and downstream of the PAM site. The comparison of the percent methylation observed at the localized CGI region for the CRISPR-edited animal (Fig. 4c) and the unedited control animal (Fig. 4d) demonstrate the introduced methylation variance as a result of the introduction of the donor DNA. Blue squares (■) indicate the percent differences in CpG methylation for HDR1 Animal from Control 2 Animal at given chromosome locations. Red diamonds (♦) indicate the percent differences in CpG methylation for Control 1 Animal from Control 2 Animal at given chromosome locations

Closer observation of the methylome at the edited CGI locations for the edited animals as compared to the control demonstrates the significant deviation in the methylation patterns for the HDR modified CGIs. Using locations with a minimum of five calls for each CpG, Fig. 5a shows the complete modification of the edited CGI (Chr15:11,035,894–11,038,084) in the experimental animal (blue squares) beyond the anticipated 20% biological noise seen in the Control 1 Animal (red diamonds). The incorporation of the donor DNA homology arms replaced the entire CGI genomic range (gold bar) on each side of the CRISPR-targeted PAM site (dashed line). Expanding beyond the genomic region influenced by the homology arms of the donor DNA, Fig. 5b shows the profile of methylation difference flanking the affected CGI by 7000 bp upstream and downstream. These results demonstrate the increase in methylation at the edit site as compared to the flanking genomic regions. The observed methylation pattern for the CGI of the CRISPR-edited HDR1 and the Control 1 are illustrated in Fig. 5c and d, respectively. The edited CpG sites of HDR1 have elevated methylation percentages as compared to that of Control 1.

**Fig. 6:**
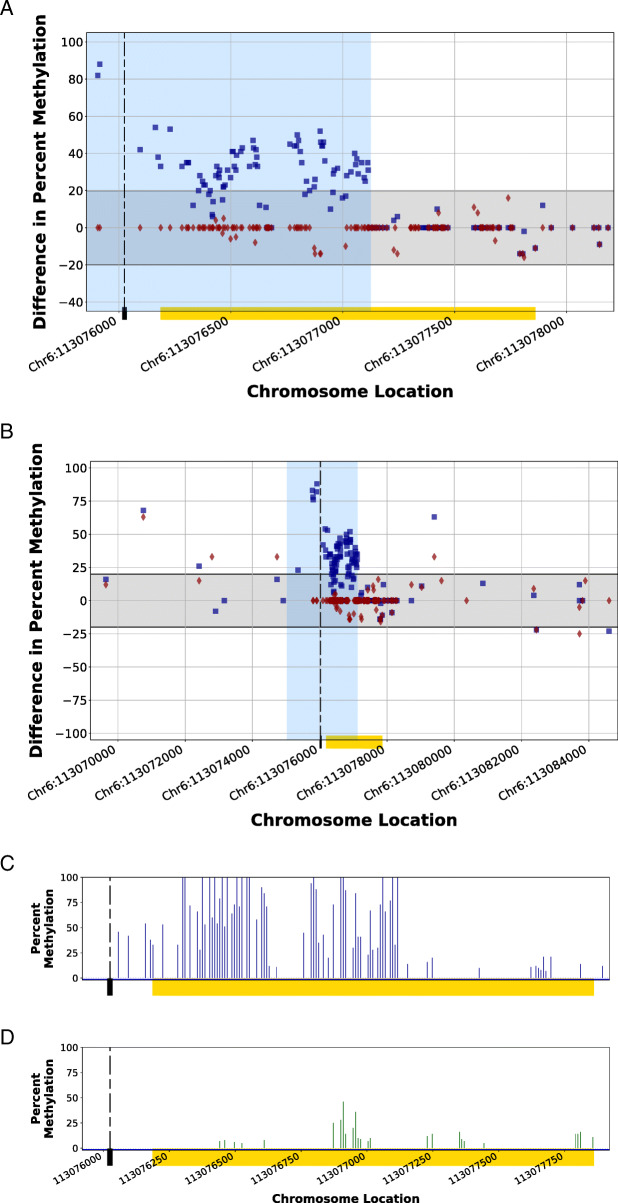
Methylomic difference of a partially edited CpG island within the genome of the HDR2 Animal using homology-directed repair in a CRISPR-mediated edit. The downstream homology arm of a synthetic DNA fragment (region shaded in blue) in a homology-directed repair of a CRISPR-mediated genomic cut spanned a portion of a CpG island (CGI; Chr6:113,076,186–113,077,861; yellow bar) within the genome of the HDR2 Animal, resulting in a destabilized methylation pattern (blue squares) as compared to the Control 1 Animal methylation patterns at the same location (red diamonds). The regions with methylation patterns modified by the incorporation of homology arms within the CGI and upstream of the CGI are shaded in a light blue background. While Fig. 5a illustrates the localized methylation variance introduced at the CGI, Fig. 5b illustrates the comparison of the modified region to flanking endogenous genomic regions by displaying the variance of methylation patterns for 7000 bp upstream and downstream of the CGI. The methylation patterns of the unmodified downstream portion of the CGI (unshaded) for the edited CGI as well as the flanking regions of endogenous genome displayed a methylation pattern similar to the control. The gray band indicates considered biological epigenetic variance (+/− 20% change). The dashed line indicates the protospacer adjacent motif (PAM) location of the targeted CRISPR cut site. The comparison of the percent methylation of the CpG sites observed at the localized CGI region for the CRISPR-edited animal (Fig. 5c) and the unedited control animal (Fig. 5d) demonstrates the introduced methylation variance as a result of the adoption of the donor DNA. Blue squares (■) indicate the percent differences in CpG methylation for HDR2 Animal from Control 2 Animal at given chromosome locations. Red diamonds (♦) indicate the percent differences in CpG methylation for Control 1 Animal from Control 2 Animal at given chromosome locations

Figure 6a illustrates the effects of the partial modification of an edited CGI (Chr6:113,076,186–113,077,861; gold bar). In this edit, the CRISPR-targeted PAM site (dashed line) is located upstream of the affected CGI. During the HDR, only the downstream homology arm was incorporated into the affected CGI range, stretching approximately half the length of the CGI (blue background shading). The resulting methylation pattern perturbation is observed in this region of the CGI only. The downstream portion of the CGI methylome (unshaded background) remains unmodified from that of the control animal. Figure 6b shows the methylation difference in the genomic regions flanking the edited CGI. Increases of methylation are concentrated at the site of homology arm interaction with the genome. The methylation percentage observed for CRISPR-edited HDR2 (Fig. 6c) and Control 1 (Fig. 6d) demonstrate the elevated methylation of the HDR2 CGI with a distinct border of methylation inflection at the interaction site of the terminus of the downstream homology arm.

**Fig. 7:**
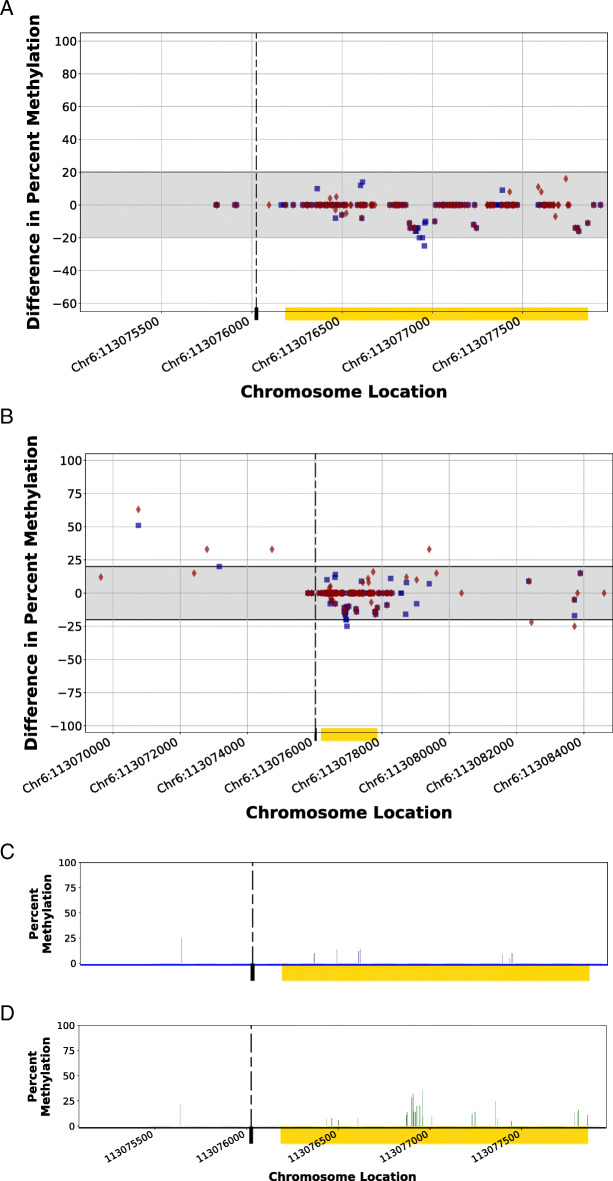
Methylomic variance of a CpG island within the genome of the NHEJ1 Animal flanking a non-homologous end-joining repair in a CRISPR-mediated edit. A non-homologous end-joining repair of a CRISPR-mediated genomic cut flanking a CpG island (CGI; Chr6:113,076,186–113,077,861; yellow bar) was inserted within the genome of the NHEJ1 Animal, resulting in a methylation pattern of the edited animal (blue squares) that was similar to that of the Control 1 Animal at the same location (red diamonds) localized around the CGI edit site (Fig. 6a) as well as 7000 bp upstream and downstream of the edit (Fig. 6b). The gray band indicates considered biological epigenetic variance (+/− 20% change). The dashed line indicates the protospacer adjacent motif (PAM) location of the targeted CRISPR cut site. The comparison of the percent methylation of the CpG sites observed at the localized CGI region for the CRISPR-edited animal (Fig. 5c) and the unedited control animal (Fig. 5d) demonstrates a lack of variance in methylation pattern as a result of non-homologous end-joining insertion of the 48-bp duplex DNA oligo fragment. Blue squares (■) indicate the percent differences in CpG methylation for NHEJ1 Animal from Control 2 Animal at given chromosome locations. Red diamonds (♦) indicate the percent differences in CpG methylation for Control 1 Animal from Control 2 Animal at given chromosome locations

In contrast, the edited CGI using NHEJ repair to insert donor DNA retains a methylation pattern that is very similar to the Control 1 Animal, demonstrating that the lack of replacement of the edited CGI (Chr6:113,076,186–113,077,861; gold bar) with exogenous insert homology arms leave the CGI methylation pattern unaffected (Fig. 7a). The NHEJ repaired CGI region is similar to that of the Control 1 Animal, supporting the CGI falling into the Case 4 category of CGIs in the statistical workflow (Table 2). Similarity between NHEJ1 and Control 1 was seen at an expanded view of the genomic region that included 7000 bp upstream and downstream of the CGI (Fig. 7b). The comparison of observed methylation percentage for NHEJ1 (Fig. 7c) and Control 1 (Fig. 7d) showed minimal difference. These observations were reproduced in comparisons of the edited CGIs of modified animals to the unedited CGIs of Control 2 Animal.

### CGIs with statistically relevant methylation changes

CGIs containing statistically significant change from that of the control animal and containing changes beyond those considered biological noise were categorized as Case 1 CGIs within the statistical workflow. Case 1 data presented within tables are a result of comparisons between CRSPR-edited or unedited animals to Control 1 Animal; for all evaluations, similar results were observed in comparisons to Control 2.

**Fig. 8:**
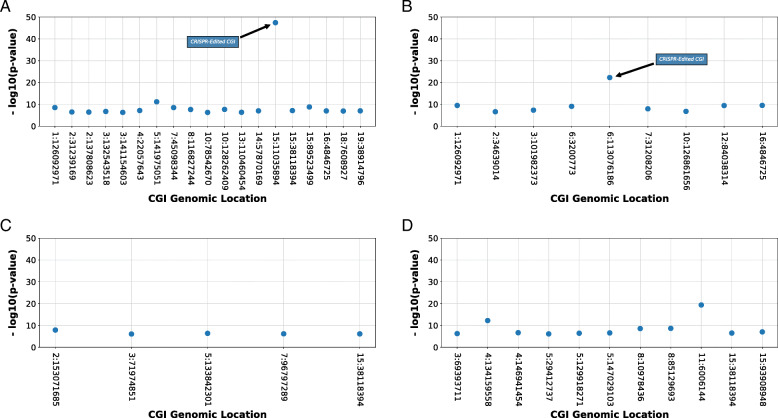
Edited CGIs affected by HDR show statistically significant *p*-values. In evaluating the methylation variance between CRISPR-edited and control mouse epigenomes, top CGIs for each comparison were determined by ranking of *p*-values observed and removing those CGIs that did not display greater than 50% of observed CpGs with methylation increases above 20%. Those top CGIs containing HDR edits had more significant *p*-values for methylation increase above biological variance (20% change) as compared to other CGIs across the mouse genome for HDR1 Animal (**a**) and HDR2 Animal (**b**). A CGI with a complete modification of its methylome displayed a significant increase of *p*-value over the control (**a**), while a CGI with only a partial modification displayed an increase of *p*-value over the control at a lesser significance (**b**). The top CGIs with methylation change within NHEJ1 Animal containing a NHEJ edit had no significant increase of *p*-value above biological variance, and the edited CGI was not among the top CGIs with methylation change (**c**). Comparison of CGIs in unedited Control 2 Animals displayed a low significance of change for the top CGIs (**d**)

Organized in smallest to largest order of *p*-value, the CRISPR-edit sites using HDR in the HDR1 Animal (3.852E-48) and the HDR2 Animal (5.384E-23) rank as the most significantly methylated CGIs within the genomes of the experimental animals as compared to the Control 1 Animal (Tables 3 and 4; Fig. 8a and b).

**Table 3:** Case 1 *p*-value rankings for HDR1 Animal compared to Control 1 Animal

					Degree of Experimental Methylation Level Higher Than Control					
Chromosome	CpG Island Start Location	CpG Island End Location	*p*-value	CpG Count	1–20% Proportion	1–20%	21–40%	41–60%	61–80%	81–100%
15	11,035,894	11,038,084	3.852E-48	91	0.034	3	9	26	45	6
5	141,975,051	141,975,377	6.732E-12	23	0.043	1	10	10	2	0
15	89,523,499	89,524,359	1.555E-09	62	0.388	19	21	8	1	0
1	126,092,971	126,093,200	2.973E-09	16	0.125	2	5	8	1	0
7	45,098,344	45,098,676	2.978E-09	15	0.000	0	0	4	7	3
10	128,262,409	128,262,692	2.055E-08	12	0.083	1	10	1	0	0
8	116,827,244	116,827,574	2.268E-08	13	0.077	1	1	9	2	0
4	22,057,643	22,057,924	6.841E-08	11	0.000	0	10	1	0	0
15	38,118,394	38,118,655	7.345E-08	16	0.250	4	11	1	0	0
19	38,914,796	38,915,109	9.360E-08	12	0.083	1	7	4	0	0

The *p*-value represents the upper-tail *p*-value for the hypothesis test of higher methylation in the experimental mouse than the control mouse. The CG count includes the total number of CGs in the CpG island. The proportion and category counts only refer to the number of CGs where the experimental CG methylation level is higher than the control CG methylation level

**Table 4:** Case 1 *p*-value rankings for HDR2 Animal compared to Control 1 Animal

					Degree of Experimental Methylation Level Higher Than Control					
Chromosome	CpG Island Start Location	CpG Island End Location	*p*-value	CpG Count	1–20% Proportion	1–20%	21–40%	41–60%	61–80%	81–100%
6	113,076,186	113,077,861	5.384E-23	135	0.229	19	45	19	0	0
16	4,846,725	4,846,950	2.937E-10	12	0.000	0	1	10	1	0
1	126,092,971	126,093,200	3.224E-10	16	0.313	5	11	0	0	0
12	84,038,314	84,038,998	3.593E-10	37	0.406	13	14	5	0	0
6	3,200,773	3,201,615	8.468E-10	35	0.355	11	20	0	0	0
7	31,208,206	31,208,620	1.111E-08	24	0.455	10	11	1	0	0
3	101,982,373	101,982,595	4.617E-08	27	0.480	12	9	4	0	0
10	126,861,656	126,861,911	1.620E-07	15	0.200	3	7	5	0	0
2	34,639,014	34,639,339	2.409E-07	22	0.400	8	10	2	0	0

The *p*-value represents the upper-tail *p*-value for the hypothesis test of higher methylation in the experimental mouse than the control mouse. The CG count includes the total number of CGs in the CpG island. The proportion and category counts only refer to the number of CGs where the experimental CG methylation level is higher than the control CG methylation level

The distribution counts of the degree of CpG methylation changes within the Case 1 CGIs demonstrates an increase of methylation toward complete methylation change at modified CpG locations, after greater than ten generations of progeny inheritance. These data highlight the generational persistence of the epigenetic changes at the edit sites within the progeny. The degree of methylation distribution is skewed toward the higher percentage of increased methylation in the HDR1 Animal at the region of the edit due to complete CGI methylome modification; while, the degree of methylation distribution is skewed toward the higher percentage of increased methylation in the HDR2 Animal, it is to a lesser degree than the HDR1 Animal due to the incompletely modified CGI. The genetically-edited CGI of the NHEJ1 Animal using NHEJ repair to introduce an indel did not rank within the most significant Case 1 CGIs for the animal (Table 5), instead the edited CGI ranking within the Case 4 category of the comparisons. The Case 1 CGIs of the NHEJ1 Animal were similar in their measure (Fig. 8c). Supplemental Figures A and B illustrate the profiles of the top two Case 1 CGIs for the NHEJ1 Animal, indicating a non-uniformed distribution of methylation increase unlike the more uniformed increase displayed in the HDR edited sites (Figs. 5 and 6).

**Table 5:** Case 1 *p*-value rankings for NHEJ1 Animal compared to Control 1 Animal

					Degree of Experimental Methylation Level Higher Than Control					
Chromosome	CpG Island Start Location	CpG Island End Location	*p*-value	CpG Count	1–20% Proportion	1–20%	21–40%	41–60%	61–80%	81–100%
2	153,071,685	153,072,285	1.222E-08	53	0.455	20	18	6	0	0
5	133,842,301	133,842,658	3.997E-07	9	0.000	0	6	3	0	0
7	96,797,289	96,797,521	6.555E-07	10	0.300	3	7	0	0	0
15	38,118,394	38,118,655	7.027E-07	9	0.000	0	5	4	0	0
3	71,974,851	71,975,406	8.074E-07	6	0.000	0	6	0	0	0

The *p*-value represents the upper-tail *p*-value for the hypothesis test of higher methylation in the experimental mouse than the control mouse. The CG count includes the total number of CGs in the CpG island. The proportion and category counts only refer to the number of CGs where the experimental CG methylation level is higher than the control CG methylation level

Evaluating the Case1 results of Control 2 compared to Control 1, the top ranked CGI (Chr11:6,006,144–6,007,748) has a skewed distribution as in the HDR1 Animal completely edited CGI. Further, the comparison of the CGI methylation changes between Control 1 and Control 2 (Table 6) highlights a significantly methylated CGI (Chr4:134,159,558–134,164,691) with similar *p*-values to those observed in the partially edited CGI of the HDR2 Animal (Tables 4 and 5; Fig. 8b and d), reflecting not only the common origins of the strains and also the more than 250 generations of reproductive separation. One significant difference between the significantly methylated CGIs of the control comparisons compared to those of the homology-directed repair edited animal is the consistency of the CpG methylation across the CGI. When visualized, the methylation patterns of the CGIs between Control 1 and Control 2 (Table 6) are inconsistent with CpG methylation calls rising and falling irregularly across the CGIs. In contrast, the methylation patterns of the edited sites display a uniformed increase of methylation across the range of the incorporated homology arm (Figs. 5 and 6).

**Table 6:** Case 1 *p*-value rankings for Control 2 Animal compared to Control 1 Animal

					Degree of Experimental Methylation Level Higher Than Control					
Chromosome	CpG Island Start Location	CpG Island End Location	*p*-value	CpG Count	1–20% Proportion	1–20%	21–40%	41–60%	61–80%	81–100%
11	6,006,144	6,007,748	4.011E-20	27	0.000	0	1	4	14	8
4	134,159,558	134,164,691	5.662E-13	28	0.179	5	14	7	2	0
8	85,129,693	85,132,058	2.147E-09	10	0.000	0	7	3	0	0
8	10,978,436	10,978,759	2.607E-09	12	0.167	2	10	0	0	0
15	93,908,948	93,909,153	8.615E-08	10	0.000	0	9	1	0	0
4	146,941,454	146,942,785	2.027E-07	79	0.321	18	25	13	0	0
5	147,029,103	147,029,460	2.538E-07	15	0.467	7	7	1	0	0
15	38,118,394	38,118,655	3.010E-07	10	0.000	0	9	1	0	0
5	129,918,271	129,918,590	3.579E-07	14	0.286	4	5	5	0	0
3	69,393,711	69,394,087	5.160E-07	13	0.231	3	10	0	0	0

The *p*-value represents the upper-tail *p*-value for the hypothesis test of higher methylation in the experimental mouse than the control mouse. The CG count includes the total number of CGs in the CpG island. The proportion and category counts only refer to the number of CGs where the experimental CG methylation level is higher than the control CG methylation level

These findings indicate that the cellular fluctuation of the methylome due to varying biological processes of time between the compared animals produce statistically significant methylation changes within CGIs between the animals, reinforcing the need to understand additional confirmatory information about the top ranking Case 1 CGIs to conclusively identify an HDR-edited CGI. Accompanying information could include large and small rearrangements, including insertion, deletion, or altered base pairs [32].

While the HDR modified CGIs were identified as containing statistically changed methylation patterns as anticipated, both the NHEJ and control comparisons identified statistically changed methylation patterns as well. These observations highlight the need to understand where edits have been directed through other indicators such as direct sequencing to confirm an HDR-directed genome modification through methylome evaluations. Using perturbations of CGI methylation patterns as indicators of genomic insertions allows for a measurable factor for indicating a genetic modification, including CRISPR-mediated HDR genomic edits.

## Discussion

The application of CRISPR approaches for the modification of the genome offers a level of control over genomic editing processes that can cure diseases, revive ecosystems, and enhance agricultural crops and livestock. Advances in these technologies are changing the approaches and strategies in molecular biology to modify and regulate genomic information, generating genomic modifications with targeted precision and specificity at an unprecedented pace. Two commonly utilized mechanisms for introducing genome modifications at CRISPR cut sites are non-homologous end-joining (NHEJ) and homology-directed repair (HDR). NHEJ is commonly used to create indels leading to gene frameshifts or loss of coding regions and inactivation of a targeted gene [3] and can also be used to insert donor DNA in the genome [33]. In practice, this approach is less precise, resulting in indels and is prone to serendipitous outcomes including hypomorphs. With HDR, the cell utilizes added donor DNA as a repair templated for insertion and genomic modification, relying on genomically homologous flanking regions in the donor sequence and the endogenous DNA repair mechanisms of the cell to incorporate it into the genome [3, 14, 15]. The addition of directed genomic modification by HDR, mediated via CRISPR, allows for the precise incorporation of any number of genomic modifications from single point mutations to whole gene insertions to chromosomal rearrangements. The findings of this study highlight the utility of using methylome perturbances induced by the insertion of donor DNA sequence in HDR mechanisms as an indicator of a directed genomic edit. With the expanding advantages of CRISPR technologies for molecular modification and observation, understanding the limiting or confounding factors of the technology is becoming increasingly important, especially in relation to the required fidelity of gene therapy applications. In parallel, the drive for therapeutic success that can in application affect the germline of a given organism also requires the development of methods for detecting and tracking induced changes within the genome.

Within the mammalian genome, CpG islands (CGIs) are regions of dense clusters of CpG dinucleotides that are stably maintained typically in a hypomethylated state [34]. Frequently located within promoter, enhancer, or gene regions, CGIs play an important regulatory role for controlling expression levels of genes and gene variants within the genome. Throughout the life of the animal, the spatial and temporal modifications of CGI methylation patterns across the genome are critical for tissue-specific differentiation and development [21]. The cellular regulatory maintenance of CGI methylation patterns in an unmethylated state provides opportunity to assess induced perturbances of the anticipated methylation pattern by HDR, appearing as epigenetic scars on the genome. Within this study, these CpG-rich segments of the mouse genome were identified using an computational search algorithm governed by the parameters of the definition of a CGI as reported by Gardiner-Garden and Frommer [18]. Using the algorithm, we identified 90,953 regions across the mouse genome with elevated CpG content (S1 Table) that we termed as CGIs. This observation is higher than other studies that have reported CGI observations in the mouse genome ranging in counts of 15,500 [35], 20,500 [36], and 37,000 [37] to name a few. Often these studies remove duplicate genome regions to adjust counts of these CpG-rich locations. Our definition focused on an inclusive algorithm with more wholistic parameters as to not exclude any regions that may fall within the definition of a CGI but may not serve a regulatory function within the genome or may be found in duplicate within the genome. Therefore, our definition serves less as a method of characterizing a functional role for the identified CGIs rather than as indicators of conserved methylation patterns located within the genome.

For the CpG locations of the defined CGIs within the study, our evaluations focused on CGIs with an increased methylation profile at their CpG locations as compared to control animals, requiring a minimum of five bisulfite sequence calls at each CpG location for increased confidence in the downstream statistical assessments for determining and evaluating the edited CGIs. While observations were made requiring a minimum as low as two and as high as twenty bisulfite sequence calls at each CpG location, no loss of information was observed by increasing the minimum to as high as ten calls.

In the ranking of significantly changed CGIs as compared to those of the control animal across the genome (Tables 3, 4, 5, and 6), corrected significance levels (α*) for hypothesis testing using the Bonferroni method increased the stringency of each individual CGI test and the resulting significant *p*-values were ranked by the smallest value indicating the CGI with the most significant change. Further, a greater than 20% methylation increase for more than 50% of the observed CpG locations within each CGI was required to account for observed biological variance. The increase of methylation within the top ranked CGIs as indicated by the *p*-values highlighted both of the HDR modified CGIs as containing the most significantly changed methylation profiles for their respective comparisons, targeting CGI candidates for deeper observation of the methylation patterns displayed within them. In contrast, the NHEJ modified CGI of the NHEJ1 Animal did not display a significant change in methylation pattern compared to the control animal and was not observed in the top ranking changing CGIs, reflecting the lack of integration of donor DNA within the genomic sequence of the edited CGI.

The dynamic nature of methylation changes within CGIs to control gene expression levels in response to stimuli such as environmental and developmental variables results in natural fluctuations of CGI methylation profiles. Comparison of genomic CGI methylation profiles of two control animals within this study (Table 6) indicated a significantly modified CGI with a *p*-value resembling that of the partially edited CGI (Table 4) and methylation distribution counts resembling that of the completely edited CGI (Table 3). These findings emphasize that ranking of changed CGIs using the methodology described in this study for the purposes of indicating HDR modified CGIs in a de novo manner would require additional validating indicators to definitively assert that a given CGI had been purposefully modified using a CRISPR-targeted HDR mechanism. Our methodology filters the CGIs to a more manageable number for further investigation. For example, instead of individually looking at the methylation pattern of 71,120 CGIs, this approach reduced the number to nine CGIs for further investigation using additional indicators. Such additional indicators might include direct sequence modifications (e.g., indels or nucleotide substitutions) or unexpected changes in transcription levels for a gene that might be under regulation by the affected CGI.

In the evaluation of the CRISPR-edited CGIs, the methylome observation at CpG locations within the targeted CGIs were bisulfite sequenced at levels above that of the overall respective genome-wide CpG bisulfite sequences (Fig. 3). These observations increase the confidence in the statistical evaluations for the CpGs of the edited CGIs.

CRISPR-mediated genome modification using HDR mechanisms to incorporate novel sequence into the genome of the mouse zygote resulted in observable and persistent destabilization of the modified methylation patterns of the CGIs affected by the donor DNA (Figs. 5 and 6). In both edits, a marked increase of methylation is observed within the affected CGIs for the CpG locations spanning the range of genomic incorporation of the donor DNA. The animals evaluated were at least 10 generations from the original founder animal showing that these CpG nucleotide methylation changes persist over generations as an echo or scar of the introduced novel genomic edit. The same observable modification in the CGI methylation pattern did not occur in the CRISPR-mediated 48-base insertion leveraging the NHEJ repair mechanisms (Fig. 7). In the mechanics of the edit, the NHEJ edit did not replace the genomic sequence of the CGI with donor DNA recombination template during its application, leaving the endogenous methylation pattern unmodified. The unmodified methylome observed in the NHEJ-edited CGI indicates that this type detection method would not detect an edit facilitated by NHEJ as being introduced using CRISPR technology. Other genomic scaring indicators would be necessary to specify the blunt-end repair editing method. In this study, the integrated fragments in the mice modified by HDR are larger (11 kb and 2005 bp) than the integrated fragment in the mouse strain modified by NHEJ (48 bp).

Takahashi, et al. [38], reported in 2017 that the integration of CpG-free DNA into CGI regions of human pluripotent stem cells induced de novo methylation of the modified CGIs. The observed de novo methylation had an absolute requirement for the integrated DNA to be free of CpGs with DNA fragments containing CpGs resulting in no change in methylation. In contrast, the CGI methylation observed in Fig. 5 and Fig. 6 are not the result of CpG-free DNA integration and map closely to the homology arms of the donor DNA. As in the study by Takahashi, et al., the findings described here point similarly to the potential introduction of epimutations into the genome during the implementation of CRISPR genome modifications.

Detecting or directing the modification of the methylome using CRISPR technology has implications in mitigating disease states, controlling gene expression, and determining when genetic modifications have been introduced into the genome. It has been reported that using CRISPR to modify the methylome patterns of the cell has impact on cellular function [33, 39]. The methylation of CpG locations within the genome is one mechanism for controlling the degree of gene expression at the cellular level, providing variable and tunable gene expression profiles across different tissue types [40, 41]. This control of gene expression allows for the genome to conduct tissue-specific functions and to respond to environmental influences [41, 42]. Disruptions of these methylation patterns within specific regions of the genome have been associated with disease states such as cancer and neurological disorders [43]. For example, in the case of imprinting, methylation of a given allele of a gene from either the maternal or paternal strand can silence the given allele for the lifetime of the cell [44]. The human diseases of Angelman syndrome, Prader-Willi syndrome, Beckwith-Wiedemann syndrome, and certain cancers are affected by paternal imprinting [45]. In these cases, the parentally inherited shutdown of a gene may allow expression of a defective gene or result in the silencing of both gene copies through uniparental disomy, resulting in disease [45]. Application of CRISPR technology to modify the epigenetic imprint of an entire gene or its promoter region may provide a technique for rescuing these type genetic disease states [33, 46]. In similar application, Alexander, et al., recently reported the silencing of the *HPRT1* housekeeping gene for cellular purine recycling by replacing a CpG island with in vitro methylated alleles of the CGI using dual cut NHEJ editing [33]. From their results, they concluded that the methylation of elements in the promoter region of a gene can functionally silence the expression of the gene.

The expansion of technical methodologies and the opening of application spaces for CRISPR genome editing facilitates advancements in synthetic biology and pathways to basic discoveries and new clinical therapies. In parallel, the potential costs of introducing disease states, environmentally toxic influences, and other concerning biological alterations of consequence call for CRISPR detection mechanisms that follow the technical progression of CRISPR modalities. The potential effects of inadvertently or intentionally inserting a genomic edit into a genome for directed modification purposes implies the need for the parallel development of equally diverse sets of methods for detection of these edits. The methodology described here represents one approach to detecting genomic scars left behind during the application of CRISPR, and we would suggest similar observations for other genomic modification approaches.

## Conclusions

In this study, we demonstrate that using CRISPR editing for the insertion of donor DNA using HDR modifies the epigenetic profile of the targeted region, increasing the degree of methylation observed at local CGIs, replaced with donor DNA. Further, these edits and the methylation changes persist over generations within the progeny of edited individuals. In this way, making genome edits with CRISPR technology at regions of importance to regulation of transcriptional gene expression may modify the epigenetic profile of genes and their regulators of expression, affecting the expression profile of the gene or genes in the vicinity. In developing strategies for genomic modifications, consideration should be given to the induced modification of the epigenetic profile that can display inheritable persistence across generations. These types of applied CRISPR-mediated epigenome edits may represent a means to correct epigenetic disease variants like genomic imprinting diseases. Further, the modification of the methylome pattern represents a genomic scar caused by the application of a directed CRISPR cut and subsequent incorporation of donor DNA into the genome. While additional corroborating information is needed to confirm these effects, our methodology highlights a supporting piece of evidence to indicate the introduction of a CRISPR-mediated genome modification using homology-directed repair.

## Methods

### Animal strains

Frozen mouse spleen tissue samples were provided by Dr. Michael V. Wiles of *The Jackson Laboratory*, Bar Harbor, ME, USA. The epigenomes of four CRISPR-mediated insertion strains and two genetically matched control strains were evaluated using bisulfite sequencing methods. Unedited control mouse strains C57BL/6 J (JAX Stock Number 000664) as Control 1 and C57BL/6NJ (JAX Stock Number 005304) as Control 2 were used. Genetically-modified strains used for this study are described in Table 1, abbreviated here as HDR1, HDR2, and NHEJ1. Mice were group housed in a climate-controlled facility with a 12-h light/dark cycle and free access to food and water. This study was conducted in accordance with the recommendations in the *Guide for the Care and Use of Laboratory Animals* [47]. All animal protocols were reviewed and approved by The Jackson Laboratory Animal Care and Use Committee (Summary #11006). Animals were euthanized by cervical dislocation and all efforts were made to minimize suffering.

### Tissue processing

For replicate animals for each strain, genomic DNA (gDNA) was extracted from excised spleen tissues of the respective strains and processed for overall genomic methylation patterns. For each animal, 5 mg of spleen tissue were ground by mechanical disruption using liquid nitrogen grinding. gDNA was isolated using a modified protocol of the Genomic DNA Isolation Kit (AbCam; #ab65358). Using 35 μL of cell lysis buffer, the pulverized tissue was washed by centrifugation. The supernatant containing cellular debris was discarded, and the resulting pellet of isolated nuclei was suspended in 40 μL of cell lysis buffer. Kit enzyme mix (5 μL) was added to the solution, mixed by gentle pipetting, and incubated in a 55 °C water bath for 1 h. gDNA was isolated from the extraction mixture using a modified protocol of the paramagnetic bead DNA isolation in the ChargeSwitch gDNA Micro Tissue Kit (Thermo Fisher Scientific; #CS11203). 1 ml ChargeSwitch Lysis Buffer (L15) and 10 μL of proteinase K were added to the nuclei lysate. The mixture was incubated in a 55 °C water bath for 1 h. After incubation, 5 μL of RNase A was added to the lysate and homogenized by pipetting. The mixture was incubated at room temperature for 5 min. gDNA was isolated following the DNA binding, DNA washing, and DNA elution processes described by the manufacturer protocol. The elution volume was concentrated using a Savant DNA120 SpeedVac Concentrator (Thermo Fisher Scientific) to a volume of 100 μL. The concentration of recovered gDNA for each sample was measured using the Qubit 3.0 fluorometer system (Thermo Fisher Scientific). Quality of each sample was evaluated using a DNA 12000 Chip Kit (Agilent; #5067–1508) on a 2100 Bioanalyzer System (Agilent; #G2939BA).

Bisulfite conversion and sequencing was performed by Zymo Research, using a Methyl MaxiSeq service. Briefly, methyl-seq libraries were prepared using 500 ng of genomic DNA, fragmented with 2 units of dsDNA Shearase Plus (Zymo Research; #E2018–50). The resulting genomic fragments were end-blunted and the 3′ terminus was modified with an adenine extension. The fragments were subsequently purified using the DNA Clean & Concentrator-5 kit (Zymo Research; #D4003). A-tailed genomic fragments were ligated to pre-annealed adapters containing 5′-methylcytosine in place of cytosine. Adapter-ligated fragments were repaired to complete duplex strands. Bisulfite conversion of the fragments was performed using the EZ DNA Methylation—Lightning kit (Zymo Research; #D5030). The fragments were amplified by polymerase chain reaction using adapter targeted primers with Illumina TruSeq indices. The size and concentration of the fragments were confirmed using an Agilent 2200 TapeStation. Methylome fragments were sequenced on an Illumina HiSeq instrument, generating paired-end 150-bp read lengths. The whole genome bisulfite sequencing technique was used to provide a genome-wide overview of CpG methylation patterns for interactions of Cas9 across the genome and observations of the modified methylation patterns of the edit site.

### Bioinformatic processing of methylome sequences

Both 5-methylcytosine and 5-hydroxymethylcytosine are detected by bisulfite treatment; however, the ratios between the two are not distinguished [48]. DNA methylation within this study was defined as a combination of both genomic methylation variants. Computational post-processing of methylome DNA sequence reads produced through bisulfite sequencing were organized and binned into paired, sample-specific files according to multiplex identifier (MID) sequences. The paired files were evaluated for sequence quality using Trimmomatic [49] in paired-end mode. Low quality sequences, sequencing artifacts, sequencing adapters, and MIDs were removed from the binned reads. The settings within the Trimmomatic program set a seed mismatch tolerance of 2, a palindrome clip threshold of 30, a simple clip threshold of 15 bp, minimal adapter length of 8 bp, Phred values set to a minimum of 10 for leading and trailing bases, a sliding window length of a minimum of 4 bp with a minimum Phred score of 15, and a minimum length of a given read set to 36 bp.

Quality-trimmed reads for each sample were aligned, respectively, in a paired-end sequence alignment to the mouse reference genome (version GRCm38.p6) using the BWA-Meth aligner [50]. The resulting sequence/alignment map (SAM) files were sorted using SAMtools sort of the SAMtools suite version 1.7 [51], converting the SAM files to sorted binary sequence/alignment map (BAM) files. Optical and library duplicates were identified and removed using Picard MarkDuplicates [52]. The deduplicated BAM files were indexed using SAMtools index [51], and depth of coverage at each region was calculated using SAMtools depth [51]. Genome-wide CpG methylation calls and evaluations were performed using MethylDackel [53]. The resulting BedGraph files were visualized using the Integrative Genomics Viewer version 2.5.2 (IGV; Broad Institute, Cambridge, MA) [54] and parsed for statistical analysis with Python version 3.8.0 [55] scripting. For each CpG dinucleotide, the estimated methylation level was obtained after merging methylated and unmethylated read counts for the cytosines. For use in statistical evaluations, the read depth for the cytosine of each CpG dinucleotide location was required to be a minimum of five.

With a Python script, CpG islands (CGIs) were defined and called within the mouse genome as previously outlined by Gardiner-Garden and Frommer [18]. The prescribed definition characterized islands greater than or equal to 200 base pairs in length with a G/C content greater than 50% and an observed to expected CpG ratio (O/E) greater than 0.6, where

$$ \mathrm{O}/\mathrm{E}=\frac{\# CpG/N}{\#C/N\times \#G/N} $$

The identified CGI genomic locations were used to filter the MethylDackel methylation calls, focusing only on CpGs located within the CGI ranges. To consolidate regions with enriched CpG content, a Python script stitched together genomic regions with CGI ranges located within 100 base pairs of each other.

### Statistical methods

Statistical analyses were leveraged to define a path to the location of a CRISPR-edited genomic site. At CGIs of the edit location, the methylation level of the experimental animal was assumed to be higher than that of the control animal due to cellular maintenance of CGIs in unmethylated states. A two-step evaluation approach was adopted to determine significant differences in increased methylation levels between the CRISPR-edited and control animals.

The first step evaluates the methylome data for genome-wide CGIs in a statistical hypothesis test of methylation level differences between the CGIs of the experimental and control animals. Since the experimental animal is anticipated to have higher methylation levels at the CRISPR edit site, an upper-tail, paired *t*-test with a significance level (α) of 0.05 was used.

The resulting hypothesis test for each CGI is:

- Ho: μ of genetically-edited animal methylation level ≤ μ of control animal methylation level
- Ha: μ of genetically-edited animal methylation level > μ of control animal methylation level

or

- Ho: μ_d_ ≤ 0
- Ha: μ_d_ > 0

Where μ_d_ is the mean methylation level difference of the genetically-edited animal methylation level from the control animal methylation level at each CpG site within the defined CGI range. The *t* statistic is calculated as:

$$ t=\frac{\overline{d}}{SE\left(\overline{d}\right)} $$

where,

$$ {x}_{i\  experimental}=\mathrm{experimental}\ \mathrm{methylation}\ \mathrm{at}\ \mathrm{CpGi} $$

$$ {y}_{i\  control}=\mathrm{control}\ \mathrm{methylation}\ \mathrm{at}\ \mathrm{CpGi} $$

$$ {d}_i={x}_{i\  experimental}-{y}_{i\  control} $$

$$ \overline{d}=\frac{\sum_{i=1}^n\left({d}_i\right)}{n} $$

$$ SE\left(\overline{d}\right)=\frac{s_d}{\sqrt{n}} $$

The *t* statistic follows a *t* distribution with n-1 degrees of freedom.

The total number of CGIs for each genetically-edited animal tested against Control 1 animal are shown in Table 2, indicating the number of *p*-value test that were conducted for each comparison. Therefore, in order to keep the overall test between animals at the 5% level of significance, the following Bonferroni correction factor was utilized, where α* equals:

$$ \upalpha \ast =\frac{\alpha }{Number\kern0.17em of\kern0.17em CGIs} $$

A second filter was then applied to the paired *t*-test results to filter biological epigenetic noise inherent to the epigenetic buffering of the transcriptome [56]. In applying this filter, CpGs within the CGI ranges were considered only where the genetically-edited animal CpG methylation was greater than the CpG methylation of the control animal. The remaining positive CpG methylation differences, resulting from the subtraction of the qualifying CpG methylation percentage of Control 1 Animal from those of CRISPR-edited animals or the Control 2 Animal, were then binned into the following categories, respective to the evaluations: ≤20%, 21–40%, 41–60%, 61–80%, and 81–100%.

If the level of increase in the genetically-edited animal at the given CpG location was less than or equal to 20%, it was considered to be biological epigenetic noise as observed across other CGIs within the genomes and not considered a significant epigenetic change. For the filter, the proportion of differences that were less than or equal to 20% was set at 0.5 across the given CGI range. This resulted in four specific categorical cases. Case 1 contains significantly increased methylation levels for the genetically-edited animal and a small degree of epigenetic noise (> 50% of CpGs with > 20% increase in methylation). Case 2 also contains significantly increased methylation levels for the genetically-edited animal but has a larger degree of epigenetic noise (< 50% of CpGs with > 20% increase in methylation). Neither Case 3 nor Case 4 contains significantly increased methylation levels for the genetically-edited animal; Case 3 has a small degree of epigenetic noise (> 50% of CpGs with > 20% increase in methylation) whereas Case 4 has a larger degree of epigenetic noise (< 50% of CpGs with > 20% increase in methylation).

### Visualizing methylation calls and variance

Initial variance at the known genomic edit sites were observed in comparisons of the sequenced methylomes of edited animals and control animals at their respective edit locations using IGV [54]. Methylation profiles from the genomic regions of the animals within the alignment BedGraph files generated by analysis in MethylDackel were mapped to the *Mus musculus* (house mouse) reference genome version GRCm38 (mm10).

With a Python script, the percent of CpG call depths were calculated for each CpG within CGIs across the genome for each animal and for CRISPR-edited CGIs for HDR1 Animal, HDR2 Animal, and NHEJ1 Animal. The percent of CpG at given call depths (1 to 10 and greater than 10) were read into a Python 3 Pandas DataFrame (version 0.25.1) [57]. The DataFrame was used as input to the Python 3 Matplotlib version 3.1.1 module pyplot [58] to generate bar plots comparing these calculated genome-wide sequence depths of CpG locations for each animal.

To relate the depth of the bisulfite sequencing coverage at each CRISPR-edited CGI to the coverage observed across all other CGIs within its respective genome, the variance observed between the methylomes of CRISPR-edited CGIs and unmodified CGIs at their CpG locations were plotted in overlaid bar plots using Pandas DataFrames and Matplotlib module pyplot. Qualifying CpGs eligible for evaluation at the CGI sites contained five calls or more at the cytidine residue. In each plot, the difference in the degree of methylation observed within the experimental animal (HDR1 Animal, HDR2 Animal, or NHEJ1 Animal) and Control 2 Animal were measured against the observed methylome of Control 1 Animal. Qualifying methylation calls were pulled from observations in BedGraph files from MethylDackel evaluations for the corresponding animals. Documented from the mouse reference genome, CpG locations within the mouse genome were recorded as a baseline for comparison against the experimental animals. For each given edited CGI, the chromosome, the CGI start location coordinate, and the CGI end location coordinate were defined to bound the data and resulting plot to the CRISPR-targeted CGI genomic location for the comparisons. The bounded data for each animal consisted of a genomic start location coordinate, a genomic end location coordinate, and observed percent methylation at the location for qualifying CpG locations within the bounded CGI genomic range. In each comparison and at each qualifying CpG location, the percent methylation values observed for the Control 1 Animal were subtracted from the qualifying CpG locations of the CRISPR-edited animal and the Control 2 Animal. Visual representations of the measured CpG methylation differences for the CRISPR-targeted CGI were illustrated using difference plots for each qualifying CpG location of each edited and control animal as compared to Control 1. Difference plots were generated as compound scatter plots using the Matplotlib module pyplot.

Manhattan plots were used to illustrate the degree of *p*-value variance observed among CGIs identified as appearing within their respective Case 1 classification and containing statistically changed methylation profiles. The calculated *p*-values for each Case 1 classified CGI within each respective comparison (HDR1 Animal to Control 1 Animal, HDR2 Animal to Control 1 Animal, NHEJ1 Animal to Control 1 Animal, and Control 2 Animal to Control 1 Animal) along with the chromosome and CGI start location coordinates were organized according to chromosome and location order in Pandas DataFrames. Using the Matplotlib module pyplot, the -log_10_ calculations of the *p*-values were illustrated as a scatter plot arranged by chromosome and location order for each comparison.

## Supplementary Information

**Additional file 1: Supplemental Table 1.** CGI algorithm statistics across the mouse genome. The CGI defining and stitching algorithm was developed and used to call CpG-rich ranges within the mouse genome. Results of the analysis provided a basis for determining targeted ranges for evaluating clustered CpG methylation variance across genomes.

**Additional file 2: Supplemental Fig. 1.** The top Case 1 CGIs identified for the NHEJ1 Animal using the statistical workflow did not correspond to the edited CGI location (Table 5). Difference profiles of the NHEJ1 Animal compared to Control 1 Animal of the two most significantly changed CGIs—Chr2:153,071,685-153,072,285 (A) and Chr5:133,842,301-133,842,658 (B)—demonstrate non-uniform methylation fluxuation in Chr2 and a change in both the Control and NHEJ1 in Chr5. In contrast, the HDR edits of Figs. 5 and 6 display uniform increase in methylation in the CpGs of the CGI above that of the Control. Blue squares (■) indicate the percent differences in CpG methylation for NHEJ1 Animal from Control 2 Animal at given chromosome locations. Red diamonds (♦) indicate the percent differences in CpG methylation for Control 1 Animal from Control 2 Animal at given chromosome locations.

## Data Availability

The datasets supporting the conclusions of this article are available in the National Center for Biotechnology Information Sequence Read Archive (SRA) under BioProject ID PRJNA676037 at https://www.ncbi.nlm.nih.gov/bioproject/676037.

## Abbreviations

BAM: Binary-sequence/alignment map
BS-Seq: Bisulfite sequencing
CRISPR: Clustered regularly interspaced short palindromic repeats
CpG: Cytosine-phosphate-guanine site
CGI: CpG island
DNA: Deoxyribonucleic acid
DNMT: DNA methyltransferase
gDNA: Genomic DNA
HDR: Homology-directed repair
IGV: Integrative Genomics Viewer
JAX: The Jackson Laboratory
M: Million
NHEJ: Non-homologous end-joining
PAM: Protospacer adjacent motif
SAM: Sequence/alignment map
