# Supplementary material for: Detection of CRISPR-mediated genome modifications through altered methylation patterns of CpG islands
Source: BMC Genomics. 2020 Dec 2;21:856. doi: 10.1186/s12864-020-07233-2 (PMC7709351; doi:10.1186/s12864-020-07233-2)
Supplement: Supplementary file 1 — Additional file 1: Supplemental Table 1. CGI algorithm statistics across the mouse genome. The CGI defining and stitching algorithm was developed and used to call CpG-rich ranges within the mouse genome. Results of the analysis provided a basis for determining targeted ranges for evaluating clustered CpG methylation variance across genomes. [file 12864_2020_7233_MOESM1_ESM.docx]

**Supplementary Table. CGI algorithm statistics across the mouse genome.**

| **Chromosome** | **Total CGIs** | **Largest CGI** | **Average CGI Length** | **Average CpG Count per CGI** | **Average GC Count per CGI** | **Average GC Composition** |
| --- | --- | --- | --- | --- | --- | --- |
| 1 | 5,990 | 5,852 | 431.26 | 26.69 | 250.13 | 53.76% |
| 2 | 6,435 | 7,072 | 461.67 | 29.68 | 271.37 | 57.48% |
| 3 | 4,835 | 10,217 | 441.07 | 27.00 | 255.41 | 55.50% |
| 4 | 6,445 | 6,093 | 446.01 | 28.38 | 261.75 | 56.11% |
| 5 | 6,910 | 9,161 | 440.00 | 27.12 | 255.13 | 55.53% |
| 6 | 4,704 | 7,188 | 448.53 | 27.73 | 260.72 | 55.82% |
| 7 | 5,576 | 6,018 | 483.04 | 30.64 | 283.80 | 56.46% |
| 8 | 5,414 | 6,505 | 448.57 | 28.01 | 261.31 | 55.63% |
| 9 | 4,675 | 5,199 | 452.92 | 28.45 | 264.09 | 55.73% |
| 10 | 5,296 | 7,334 | 426.61 | 26.09 | 246.78 | 55.43% |
| 11 | 5,518 | 8,286 | 493.21 | 32.07 | 291.34 | 56.35% |
| 12 | 4,040 | 5,685 | 443.71 | 28.07 | 258.78 | 55.69% |
| 13 | 4,292 | 6,589 | 432.85 | 26.45 | 248.58 | 55.06% |
| 14 | 3,642 | 6,099 | 447.18 | 27.54 | 259.55 | 55.57% |
| 15 | 4,004 | 4,962 | 447.41 | 28.18 | 261.46 | 55.83% |
| 16 | 3,110 | 4,491 | 435.99 | 26.74 | 251.99 | 55.46% |
| 17 | 4,306 | 5,950 | 446.74 | 28.04 | 260.84 | 55.80% |
| 18 | 3,034 | 5,331 | 442.01 | 26.98 | 254.66 | 55.19% |
| 19 | 2,727 | 7,756 | 462.12 | 29.35 | 270.60 | 55.83% |
| **Whole Genome** | 90,953 | 10,217 | 449.50 | 28.13 | 261.94 | 55.75% |

The CGI defining and stitching algorithm was developed and used to call CpG-rich ranges within the mouse genome. Results of the analysis provided a basis for determining targeted ranges for evaluating clustered CpG methylation variance across genomes.
