# Supplementary figures and images for: Detection of CRISPR-mediated genome modifications through altered methylation patterns of CpG islands
Source: BMC Genomics. 2020 Dec 2;21:856. doi: 10.1186/s12864-020-07233-2 (PMC7709351; doi:10.1186/s12864-020-07233-2)

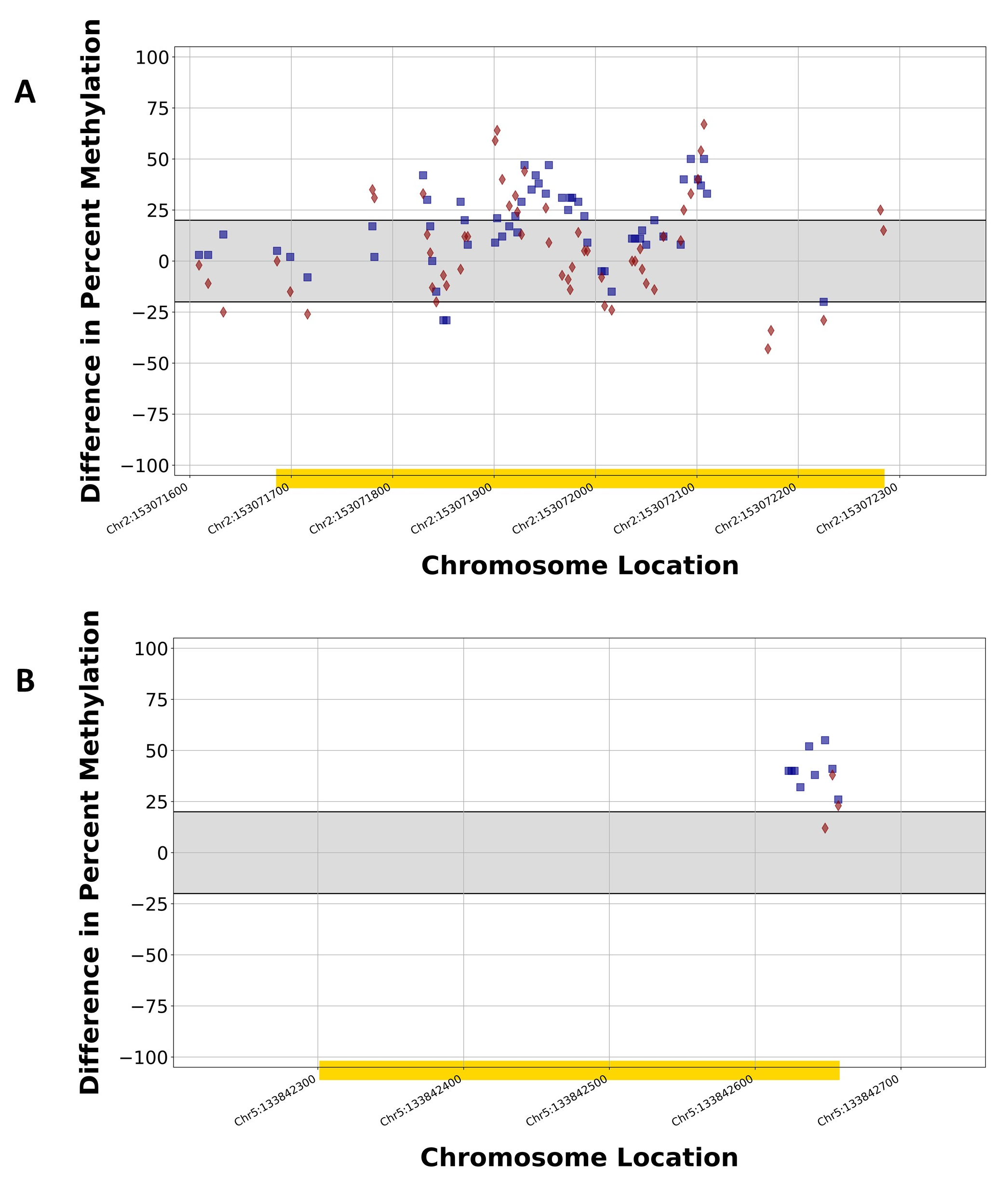

Supplement: Supplementary file 2 — Additional file 2: Supplemental Fig. 1. The top Case 1 CGIs identified for the NHEJ1 Animal using the statistical workflow did not correspond to the edited CGI location (Table 5). Difference profiles of the NHEJ1 Animal compared to Control 1 Animal of the two most significantly changed CGIs—Chr2:153,071,685-153,072,285 (A) and Chr5:133,842,301-133,842,658 (B)—demonstrate non-uniform methylation fluxuation in Chr2 and a change in both the Control and NHEJ1 in Chr5. In contrast, the HDR edits of Figs. 5 and 6 display uniform increase in methylation in the CpGs of the CGI above that of the Control. Blue squares (■) indicate the percent differences in CpG methylation for NHEJ1 Animal from Control 2 Animal at given chromosome locations. Red diamonds (♦) indicate the percent differences in CpG methylation for Control 1 Animal from Control 2 Animal at given chromosome locations. [file 12864_2020_7233_MOESM2_ESM.tif]
